# Supplementary material for: Pyruvate Dehydrogenase Kinase 1 inhibition mediated oxidative phosphorylation enhancement in cartilage promotes osteoarthritis progression
Source: BMC Musculoskelet Disord. 2023 Jul 20;24:597. doi: 10.1186/s12891-023-06585-6 (PMC10357736; doi:10.1186/s12891-023-06585-6)
Supplement: Supplementary file 1 — Additional file 1. [file 12891_2023_6585_MOESM1_ESM.zip › MF.Gsea.1653627316242/GOMF_HYDROLASE_ACTIVITY_ACTING_ON_CARBON_NITROGEN_BUT_NOT_PEPTIDE_BONDS.html]

Details for gene set GOMF\_HYDROLASE\_ACTIVITY\_ACTING\_ON\_CARBON\_NITROGEN\_BUT\_NOT\_PEPTIDE\_BONDS[GSEA]

|  || Dataset | 55457\_collapsed\_to\_symbols.cls.cls #OA\_versus\_normal.cls.cls #OA\_versus\_normal\_repos |
| Phenotype | cls.cls#OA\_versus\_normal\_repos |
| Upregulated in class | OA |
| GeneSet | GOMF\_HYDROLASE\_ACTIVITY\_ACTING\_ON\_CARBON\_NITROGEN\_BUT\_NOT\_PEPTIDE\_BONDS |
| Enrichment Score (ES) | 0.26889145 |
| Normalized Enrichment Score (NES) | 1.2425941 |
| Nominal p-value | 0.104347825 |
| FDR q-value | 0.47691607 |
| FWER p-Value | 1.0 |
Table: GSEA Results Summary

  

Fig 1: Enrichment plot: GOMF\_HYDROLASE\_ACTIVITY\_ACTING\_ON\_CARBON\_NITROGEN\_BUT\_NOT\_PEPTIDE\_BONDS      
 Profile of the Running ES Score & Positions of GeneSet Members on the Rank Ordered List

  

| SYMBOL | TITLE | RANK IN GENE LIST | RANK METRIC SCORE | RUNNING ES | CORE ENRICHMENT || 1 | ACY1 | aminoacylase 1 [Source:HGNC Symbol;Acc:HGNC:177] | 86 | 0.937 | 0.0211 | Yes |
| 2 | AGMAT | agmatinase [Source:HGNC Symbol;Acc:HGNC:18407] | 147 | 0.866 | 0.0421 | Yes |
| 3 | HINT1 | histidine triad nucleotide binding protein 1 [Source:HGNC Symbol;Acc:HGNC:4912] | 369 | 0.718 | 0.0462 | Yes |
| 4 | ATIC | 5-aminoimidazole-4-carboxamide ribonucleotide formyltransferase/IMP cyclohydrolase [Source:HGNC Symbol;Acc:HGNC:794] | 401 | 0.706 | 0.0647 | Yes |
| 5 | DPYSL3 | dihydropyrimidinase like 3 [Source:HGNC Symbol;Acc:HGNC:3015] | 461 | 0.675 | 0.0801 | Yes |
| 6 | DPYS | dihydropyrimidinase [Source:HGNC Symbol;Acc:HGNC:3013] | 509 | 0.656 | 0.0959 | Yes |
| 7 | BTD | biotinidase [Source:HGNC Symbol;Acc:HGNC:1122] | 613 | 0.619 | 0.1063 | Yes |
| 8 | DDAH1 | dimethylarginine dimethylaminohydrolase 1 [Source:HGNC Symbol;Acc:HGNC:2715] | 620 | 0.616 | 0.1241 | Yes |
| 9 | AGA | aspartylglucosaminidase [Source:HGNC Symbol;Acc:HGNC:318] | 647 | 0.606 | 0.1400 | Yes |
| 10 | NTAN1 | N-terminal asparagine amidase [Source:HGNC Symbol;Acc:HGNC:29909] | 781 | 0.574 | 0.1467 | Yes |
| 11 | NIT1 | nitrilase 1 [Source:HGNC Symbol;Acc:HGNC:7828] | 1063 | 0.503 | 0.1397 | Yes |
| 12 | DCLRE1A | DNA cross-link repair 1A [Source:HGNC Symbol;Acc:HGNC:17660] | 1073 | 0.500 | 0.1538 | Yes |
| 13 | DCTD | dCMP deaminase [Source:HGNC Symbol;Acc:HGNC:2710] | 1107 | 0.494 | 0.1659 | Yes |
| 14 | HDAC3 | histone deacetylase 3 [Source:HGNC Symbol;Acc:HGNC:4854] | 1325 | 0.454 | 0.1624 | Yes |
| 15 | APOBEC3C | apolipoprotein B mRNA editing enzyme catalytic subunit 3C [Source:HGNC Symbol;Acc:HGNC:17353] | 1355 | 0.450 | 0.1735 | Yes |
| 16 | DCLRE1B | DNA cross-link repair 1B [Source:HGNC Symbol;Acc:HGNC:17641] | 1521 | 0.426 | 0.1733 | Yes |
| 17 | DARS1 | aspartyl-tRNA synthetase 1 [Source:HGNC Symbol;Acc:HGNC:2678] | 1558 | 0.419 | 0.1829 | Yes |
| 18 | NAAA | N-acylethanolamine acid amidase [Source:HGNC Symbol;Acc:HGNC:736] | 1569 | 0.418 | 0.1945 | Yes |
| 19 | NDST2 | N-deacetylase and N-sulfotransferase 2 [Source:HGNC Symbol;Acc:HGNC:7681] | 1587 | 0.415 | 0.2055 | Yes |
| 20 | AMDHD2 | amidohydrolase domain containing 2 [Source:HGNC Symbol;Acc:HGNC:24262] | 1595 | 0.415 | 0.2173 | Yes |
| 21 | CAT | catalase [Source:HGNC Symbol;Acc:HGNC:1516] | 1731 | 0.394 | 0.2184 | Yes |
| 22 | SIRT5 | sirtuin 5 [Source:HGNC Symbol;Acc:HGNC:14933] | 1758 | 0.390 | 0.2280 | Yes |
| 23 | AMPD1 | adenosine monophosphate deaminase 1 [Source:HGNC Symbol;Acc:HGNC:468] | 1789 | 0.385 | 0.2371 | Yes |
| 24 | AICDA | activation induced cytidine deaminase [Source:HGNC Symbol;Acc:HGNC:13203] | 1825 | 0.381 | 0.2456 | Yes |
| 25 | ADARB2 | adenosine deaminase RNA specific B2 (inactive) [Source:HGNC Symbol;Acc:HGNC:227] | 1828 | 0.381 | 0.2568 | Yes |
| 26 | APOBEC3G | apolipoprotein B mRNA editing enzyme catalytic subunit 3G [Source:HGNC Symbol;Acc:HGNC:17357] | 1917 | 0.370 | 0.2609 | Yes |
| 27 | ADA2 | adenosine deaminase 2 [Source:HGNC Symbol;Acc:HGNC:1839] | 1955 | 0.367 | 0.2689 | Yes |
| 28 | ASAH1 | N-acylsphingosine amidohydrolase 1 [Source:HGNC Symbol;Acc:HGNC:735] | 2201 | 0.338 | 0.2598 | No |
| 29 | DDAH2 | dimethylarginine dimethylaminohydrolase 2 [Source:HGNC Symbol;Acc:HGNC:2716] | 2337 | 0.322 | 0.2588 | No |
| 30 | KLK3 | kallikrein related peptidase 3 [Source:HGNC Symbol;Acc:HGNC:6364] | 2627 | 0.287 | 0.2448 | No |
| 31 | APOBEC1 | apolipoprotein B mRNA editing enzyme catalytic subunit 1 [Source:HGNC Symbol;Acc:HGNC:604] | 2695 | 0.280 | 0.2478 | No |
| 32 | FHIT | fragile histidine triad diadenosine triphosphatase [Source:HGNC Symbol;Acc:HGNC:3701] | 2758 | 0.274 | 0.2511 | No |
| 33 | PADI2 | peptidyl arginine deiminase 2 [Source:HGNC Symbol;Acc:HGNC:18341] | 2810 | 0.269 | 0.2551 | No |
| 34 | NTAQ1 | N-terminal glutamine amidase 1 [Source:HGNC Symbol;Acc:HGNC:25490] | 2856 | 0.265 | 0.2595 | No |
| 35 | RIDA | reactive intermediate imine deaminase A homolog [Source:HGNC Symbol;Acc:HGNC:16897] | 3488 | 0.203 | 0.2162 | No |
| 36 | NGLY1 | N-glycanase 1 [Source:HGNC Symbol;Acc:HGNC:17646] | 3581 | 0.196 | 0.2148 | No |
| 37 | APOBEC3F | apolipoprotein B mRNA editing enzyme catalytic subunit 3F [Source:HGNC Symbol;Acc:HGNC:17356] | 3603 | 0.193 | 0.2189 | No |
| 38 | ASPA | aspartoacylase [Source:HGNC Symbol;Acc:HGNC:756] | 3610 | 0.192 | 0.2242 | No |
| 39 | SIRT3 | sirtuin 3 [Source:HGNC Symbol;Acc:HGNC:14931] | 4009 | 0.155 | 0.1977 | No |
| 40 | ASRGL1 | asparaginase and isoaspartyl peptidase 1 [Source:HGNC Symbol;Acc:HGNC:16448] | 4122 | 0.144 | 0.1932 | No |
| 41 | AMPD3 | adenosine monophosphate deaminase 3 [Source:HGNC Symbol;Acc:HGNC:470] | 4174 | 0.140 | 0.1934 | No |
| 42 | APOBEC2 | apolipoprotein B mRNA editing enzyme catalytic subunit 2 [Source:HGNC Symbol;Acc:HGNC:605] | 4279 | 0.130 | 0.1891 | No |
| 43 | PADI3 | peptidyl arginine deiminase 3 [Source:HGNC Symbol;Acc:HGNC:18337] | 4315 | 0.127 | 0.1901 | No |
| 44 | HDAC5 | histone deacetylase 5 [Source:HGNC Symbol;Acc:HGNC:14068] | 4336 | 0.125 | 0.1923 | No |
| 45 | ARG2 | arginase 2 [Source:HGNC Symbol;Acc:HGNC:664] | 4476 | 0.115 | 0.1849 | No |
| 46 | SIRT4 | sirtuin 4 [Source:HGNC Symbol;Acc:HGNC:14932] | 4477 | 0.115 | 0.1883 | No |
| 47 | APOBEC3B | apolipoprotein B mRNA editing enzyme catalytic subunit 3B [Source:HGNC Symbol;Acc:HGNC:17352] | 5089 | 0.065 | 0.1425 | No |
| 48 | HDAC9 | histone deacetylase 9 [Source:HGNC Symbol;Acc:HGNC:14065] | 5183 | 0.058 | 0.1369 | No |
| 49 | DHODH | dihydroorotate dehydrogenase (quinone) [Source:HGNC Symbol;Acc:HGNC:2867] | 5284 | 0.049 | 0.1306 | No |
| 50 | OPLAH | "5-oxoprolinase, ATP-hydrolysing [Source:HGNC Symbol;Acc:HGNC:8149]" | 5339 | 0.045 | 0.1277 | No |
| 51 | DPYSL4 | dihydropyrimidinase like 4 [Source:HGNC Symbol;Acc:HGNC:3016] | 5712 | 0.015 | 0.0990 | No |
| 52 | DPYSL2 | dihydropyrimidinase like 2 [Source:HGNC Symbol;Acc:HGNC:3014] | 5839 | 0.006 | 0.0894 | No |
| 53 | NIT2 | nitrilase family member 2 [Source:HGNC Symbol;Acc:HGNC:29878] | 6200 | -0.021 | 0.0618 | No |
| 54 | SIRT2 | sirtuin 2 [Source:HGNC Symbol;Acc:HGNC:10886] | 6279 | -0.026 | 0.0565 | No |
| 55 | TGM2 | transglutaminase 2 [Source:HGNC Symbol;Acc:HGNC:11778] | 6355 | -0.032 | 0.0516 | No |
| 56 | CAD | "carbamoyl-phosphate synthetase 2, aspartate transcarbamylase, and dihydroorotase [Source:HGNC Symbol;Acc:HGNC:1424]" | 6359 | -0.032 | 0.0523 | No |
| 57 | PDF | "peptide deformylase, mitochondrial [Source:HGNC Symbol;Acc:HGNC:30012]" | 6922 | -0.074 | 0.0106 | No |
| 58 | ADAT1 | adenosine deaminase tRNA specific 1 [Source:HGNC Symbol;Acc:HGNC:228] | 7396 | -0.110 | -0.0231 | No |
| 59 | MTHFD2L | methylenetetrahydrofolate dehydrogenase (NADP+ dependent) 2 like [Source:HGNC Symbol;Acc:HGNC:31865] | 7789 | -0.141 | -0.0495 | No |
| 60 | HDAC6 | histone deacetylase 6 [Source:HGNC Symbol;Acc:HGNC:14064] | 7833 | -0.143 | -0.0486 | No |
| 61 | HDAC1 | histone deacetylase 1 [Source:HGNC Symbol;Acc:HGNC:4852] | 7911 | -0.148 | -0.0502 | No |
| 62 | ADA | adenosine deaminase [Source:HGNC Symbol;Acc:HGNC:186] | 8105 | -0.163 | -0.0605 | No |
| 63 | VNN1 | vanin 1 [Source:HGNC Symbol;Acc:HGNC:12705] | 8314 | -0.179 | -0.0714 | No |
| 64 | CDA | cytidine deaminase [Source:HGNC Symbol;Acc:HGNC:1712] | 8324 | -0.180 | -0.0668 | No |
| 65 | HDAC4 | histone deacetylase 4 [Source:HGNC Symbol;Acc:HGNC:14063] | 8416 | -0.187 | -0.0683 | No |
| 66 | GLS | glutaminase [Source:HGNC Symbol;Acc:HGNC:4331] | 8423 | -0.187 | -0.0633 | No |
| 67 | SIRT6 | sirtuin 6 [Source:HGNC Symbol;Acc:HGNC:14934] | 8532 | -0.196 | -0.0659 | No |
| 68 | SIRT7 | sirtuin 7 [Source:HGNC Symbol;Acc:HGNC:14935] | 8733 | -0.211 | -0.0752 | No |
| 69 | PADI4 | peptidyl arginine deiminase 4 [Source:HGNC Symbol;Acc:HGNC:18368] | 8747 | -0.212 | -0.0699 | No |
| 70 | NADSYN1 | NAD synthetase 1 [Source:HGNC Symbol;Acc:HGNC:29832] | 8858 | -0.222 | -0.0719 | No |
| 71 | SIRT1 | sirtuin 1 [Source:HGNC Symbol;Acc:HGNC:14929] | 8947 | -0.227 | -0.0721 | No |
| 72 | PGLYRP4 | peptidoglycan recognition protein 4 [Source:HGNC Symbol;Acc:HGNC:30015] | 9087 | -0.239 | -0.0758 | No |
| 73 | ADAR | adenosine deaminase RNA specific [Source:HGNC Symbol;Acc:HGNC:225] | 9195 | -0.247 | -0.0769 | No |
| 74 | DPEP1 | dipeptidase 1 [Source:HGNC Symbol;Acc:HGNC:3002] | 9353 | -0.260 | -0.0814 | No |
| 75 | HDAC2 | histone deacetylase 2 [Source:HGNC Symbol;Acc:HGNC:4853] | 9359 | -0.260 | -0.0741 | No |
| 76 | GCH1 | GTP cyclohydrolase 1 [Source:HGNC Symbol;Acc:HGNC:4193] | 9465 | -0.271 | -0.0742 | No |
| 77 | CD101 | CD101 molecule [Source:HGNC Symbol;Acc:HGNC:5949] | 9511 | -0.275 | -0.0696 | No |
| 78 | AMPD2 | adenosine monophosphate deaminase 2 [Source:HGNC Symbol;Acc:HGNC:469] | 9530 | -0.276 | -0.0628 | No |
| 79 | ARG1 | arginase 1 [Source:HGNC Symbol;Acc:HGNC:663] | 9556 | -0.279 | -0.0565 | No |
| 80 | UPB1 | beta-ureidopropionase 1 [Source:HGNC Symbol;Acc:HGNC:16297] | 9609 | -0.284 | -0.0521 | No |
| 81 | VNN2 | vanin 2 [Source:HGNC Symbol;Acc:HGNC:12706] | 9658 | -0.288 | -0.0473 | No |
| 82 | FAAH | fatty acid amide hydrolase [Source:HGNC Symbol;Acc:HGNC:3553] | 9787 | -0.300 | -0.0484 | No |
| 83 | ZBP1 | Z-DNA binding protein 1 [Source:HGNC Symbol;Acc:HGNC:16176] | 10164 | -0.337 | -0.0678 | No |
| 84 | MTHFD1 | "methylenetetrahydrofolate dehydrogenase, cyclohydrolase and formyltetrahydrofolate synthetase 1 [Source:HGNC Symbol;Acc:HGNC:7432]" | 10249 | -0.346 | -0.0641 | No |
| 85 | ADARB1 | adenosine deaminase RNA specific B1 [Source:HGNC Symbol;Acc:HGNC:226] | 10295 | -0.350 | -0.0572 | No |
| 86 | APOBEC3A | apolipoprotein B mRNA editing enzyme catalytic subunit 3A [Source:HGNC Symbol;Acc:HGNC:17343] | 10550 | -0.378 | -0.0658 | No |
| 87 | MTA2 | metastasis associated 1 family member 2 [Source:HGNC Symbol;Acc:HGNC:7411] | 10600 | -0.383 | -0.0583 | No |
| 88 | VNN3 | vanin 3 [Source:HGNC Symbol;Acc:HGNC:16431] | 10721 | -0.395 | -0.0559 | No |
| 89 | NDST1 | N-deacetylase and N-sulfotransferase 1 [Source:HGNC Symbol;Acc:HGNC:7680] | 11241 | -0.462 | -0.0827 | No |
| 90 | MIER2 | MIER family member 2 [Source:HGNC Symbol;Acc:HGNC:29210] | 11375 | -0.483 | -0.0788 | No |
| 91 | PADI1 | peptidyl arginine deiminase 1 [Source:HGNC Symbol;Acc:HGNC:18367] | 11432 | -0.493 | -0.0686 | No |
| 92 | GLS2 | glutaminase 2 [Source:HGNC Symbol;Acc:HGNC:29570] | 11547 | -0.507 | -0.0624 | No |
| 93 | PGLYRP1 | peptidoglycan recognition protein 1 [Source:HGNC Symbol;Acc:HGNC:8904] | 11646 | -0.524 | -0.0545 | No |
| 94 | PIGL | phosphatidylinositol glycan anchor biosynthesis class L [Source:HGNC Symbol;Acc:HGNC:8966] | 11817 | -0.553 | -0.0514 | No |
| 95 | ACR | acrosin [Source:HGNC Symbol;Acc:HGNC:126] | 11880 | -0.567 | -0.0394 | No |
| 96 | MTHFD2 | "methylenetetrahydrofolate dehydrogenase (NADP+ dependent) 2, methenyltetrahydrofolate cyclohydrolase [Source:HGNC Symbol;Acc:HGNC:7434]" | 12239 | -0.656 | -0.0479 | No |
| 97 | CDADC1 | cytidine and dCMP deaminase domain containing 1 [Source:HGNC Symbol;Acc:HGNC:20299] | 12267 | -0.667 | -0.0302 | No |
| 98 | CRMP1 | collapsin response mediator protein 1 [Source:HGNC Symbol;Acc:HGNC:2365] | 12491 | -0.746 | -0.0255 | No |
| 99 | HDAC11 | histone deacetylase 11 [Source:HGNC Symbol;Acc:HGNC:19086] | 12601 | -0.799 | -0.0103 | No |
| 100 | HDAC7 | histone deacetylase 7 [Source:HGNC Symbol;Acc:HGNC:14067] | 12870 | -1.131 | 0.0023 | No |
Table: GSEA details [plain text format]

  

Fig 2: GOMF\_HYDROLASE\_ACTIVITY\_ACTING\_ON\_CARBON\_NITROGEN\_BUT\_NOT\_PEPTIDE\_BONDS      
 Blue-Pink O' Gram in the Space of the Analyzed GeneSet

  

Fig 3: GOMF\_HYDROLASE\_ACTIVITY\_ACTING\_ON\_CARBON\_NITROGEN\_BUT\_NOT\_PEPTIDE\_BONDS: Random ES distribution      
 Gene set null distribution of ES for **GOMF\_HYDROLASE\_ACTIVITY\_ACTING\_ON\_CARBON\_NITROGEN\_BUT\_NOT\_PEPTIDE\_BONDS**
